# Characterization of the Biosurfactant Produced by Indigenous Bacteria from Mature Fine Tailings

**DOI:** 10.3390/bioengineering13040452

**Published:** 2026-04-13

**Authors:** Shima Shojaei, Catherine N. Mulligan

**Affiliations:** Department of Building, Civil and Environmental Engineering, Concordia University, 1455 de Maisonneuve Blvd. W., Montreal, QC H3G 1M8, Canada

**Keywords:** biosurfactants, mature fine tailings, bioremediation, oil sands, anaerobic conditions

## Abstract

Biosurfactants offer a green, sustainable approach to many environmental bioremediations, especially for oil contamination. In this study, the aim is to evaluate the effectiveness of biosurfactants in accelerating hydrocarbon removal from mature fine tailings under anaerobic conditions. The bacteria were isolated from mature fine tailings and tested for biosurfactant production using different biosurfactant screening methods (i.e., blood agar, cetyltrimethylammonium bromide (CTAB) blue agar, oil displacement, and drop collapse). The most efficient strain showed high similarity to *Stutzerimonas stutzeri* by *16S rRNA* gene sequencing. Results showed that this strain produces rhamnolipids with a critical micelle concentration (CMC) of 600 mg/L and a minimum surface tension of 38.70 ± 0.08 mN/m. Moreover, when supplemented with whey, the strain showed a high emulsification index of 24 toward toluene (66%) and hexane (60%). The bioremediation of mature fine tailings (MFTs) was conducted under anaerobic conditions by adding a consortium of the four strains that were positive in biosurfactant screening tests. The results showed 53% removal of n-alkane C9-C30 and a reduction in surface tension from 69 ± 0.5 mN/m to a minimum of 54.33 ± 0.5 mN/m. The results suggest the potential successful application of bioaugmentation for in situ biological treatment in the oil sands industry.

## 1. Introduction

Mature fine tailings (MFTs) are a by-product of the oil sands industry, generated during the extraction and processing of bitumen from surface mining operations. These tailings accumulate in large reservoirs known as tailings ponds. They consist of fine particles of sand, silt, clay, residual bitumen, and complex hydrocarbons, which require significant time to settle and form mature fine tailings [1]. The hydrocarbon pollutants in MFTs primarily consist of persistent organic pollutants (POPs) and polycyclic aromatic hydrocarbons (PAHs), which pose significant risks to environmental and human health due to their toxicity, long-term persistence, and potential for bioaccumulation [2].

Given the challenges posed by these contaminants, biological treatment methods are increasingly favoured as eco-friendly solutions to remediate polluted sites. Techniques such as bioaugmentation and biostimulation are commonly used in bioremediations. Bioaugmentation involves introducing either exogenous or indigenous microorganisms to biologically remediate the pollutants. Biostimulation refers to adding nutrients (organic and inorganic) to stimulate the growth of the target microorganisms [3]. The limited availability of hydrocarbon pollutants can hinder the success of bioremediation processes. Nevertheless, introducing bacteria that produce biosurfactants may effectively improve the breakdown of these hydrocarbon contaminants [4]. Biosurfactants are amphiphilic substances that some microorganisms, such as yeast, bacteria, and fungi, secrete as secondary metabolism in response to stressful environmental conditions. These compounds enhance the emulsification properties in oil–water mixtures by reducing the surface and interfacial tension [5]. This property facilitates contact between two non-soluble liquids by creating an emulsification layer. Thus, the contaminants become bioavailable to other microorganisms that can use them as an energy source for growth. As a result, the contamination is transformed into less toxic substances or is eliminated [6]. Naturally derived biosurfactants are becoming increasingly popular compared to chemically synthesized surfactants because they are biodegradable, less toxic, and effective under harsh pH, temperature, and salinity conditions [7].

Although biosurfactant applications have been widely studied in bioremediation processes, most of them have focused on aerobic environments or strains isolated from soil or marine environments [8,9,10,11,12,13]. Previous studies have demonstrated bioremediation of the mature fine tailings occurs in anaerobic conditions [14]. Despite the environmental importance of this fact, a few studies have explored the role of biosurfactant production under anaerobic conditions, particularly produced by indigenous bacteria.

Furthermore, oil-sands-industry-generated MFTs represent a complex environment characterized by a mixture of hydrocarbons and the presence of co-contaminants that significantly influence microbial activity, contaminant bioavailability and biosurfactant effectiveness. Microorganisms native to this environment may possess unique metabolic capabilities that cannot be observed in simplified laboratory test [15,16] systems. Thus, the objective of this study is to evaluate native bacteria originating from MFTs and assess their potential for biosurfactant production; and, moreover, to evaluate the effectiveness of biosurfactant production in enhancing hydrocarbon removal from mature fine tailings (MFTs) under anaerobic conditions.

## 2. Materials and Methods

### 2.1. Sample Collection

A sample was obtained in a 20 L container from the mature fine tailings (MFTs) layer of an oil sands pond from Edmonton, Alberta. The sample was stored at 4 °C until the analysis and experiments. MFTs formed a slurry with suspended particles of 67% silt, 2% sand, 6% colloid and 25% clay with an average particle size of less than 30 µm.

### 2.2. Bacteria Enrichment

Bacterial isolation was performed using an enrichment technique. This step was performed under anaerobic conditions, which help to eliminate unwanted bacteria and stimulate the growth of target bacteria. One gram of MFTs was added to 25 mL of minimal salt media (MSM) supplemented with 1% crude oil, and the mixture was incubated for 7 days at 37 °C. The MSM combination and their concentrations are demonstrated in Table 1 [17]. The flasks were covered with aluminum foil to prevent light penetration and placed on an orbital shaker at 150 rpm. Nitrogen gas was used to maintain an anaerobic environment. The media pH was adjusted to 7 before autoclaving at 121 °C and 1 bar pressure for 45 min. After 7 days, the media was refreshed by adding 1 mL of cultivated bacteria to 25 mL of fresh MSM, and incubation continued for three more days under the same environmental conditions [18].

### 2.3. Isolation of Indigenous Bacteria

To isolate the surviving bacteria from the enrichment step, serial dilutions of the culture broth were prepared, and bacteria were inoculated onto agar plates using the spread plate method. The plates were sealed and incubated anaerobically for 3 days at 37 °C (gas and jar technique) [19]. Three different agar media supplemented with 1% crude oil were used for bacteria isolation: Bushnell–Haas agar, minimal salt agar, and R2A Difco agar R2A (Difco™) was purchased from BD (Becton, Dickinson and Company, Franklin Lakes, NJ, USA). A Bushnell–Haas agar composition for 1 L media was prepared as: magnesium sulfate (MgSO_4_) (0.2 g), calcium chloride (CaCl_2_) (0.02 g), monopotassium phosphate (KH_2_PO_4_) (1.0 g), dipotassium phosphate (K_2_HPO_4_) (1.0 g), ammonium nitrate (NH_4_NO_3_) (1.0 g), ferric chloride (FeCl_3_) (0.05 g), and agar (15.0 g). The minimal salt agar composition is listed in Table 1, which was supplemented with 1.5% (*w*/*v*) agar powder. All the chemical materials were purchased from Fisher Scientific, Ottawa, ON, Canada and were of analytical grade. Colonies showing distinct morphology were selected and sequentially streaked 3 times on Luria–Bertani (LB) agar plates with the 4-way streak method to ensure the isolation of a uniform and pure culture. LB agar were prepared by mixing tryptone (10 g/L), yeast extract (5 g/L), sodium chloride (NaCl) (10 g/L), and agar (15 g/L). The media pH was adjusted to 7 by adding 1 M NaOH or 1 N HCL, and was autoclaved at 121 °C and 1 bar pressure for 45 min. To obtain sufficient biomass, each isolate was cultured overnight on LB broth at 30 °C in a closed-capped sterile culture tube on a shaker at 150 rpm and was suspended in sterile glycerol (final concentration 20% *v*/*v*) and stored at −20 °C for future experiments.

### 2.4. Inoculum Preparation

Each colony was cultured overnight on LB broth at 37 °C. The cells were harvested with an optical density of 600 nm at 1.0 by centrifugation at 5000× *g* for 10 min. The upper layer of the test tube, which contained the culture broth (supernatant) was removed using a sterile needle and syringe. The settled cell pellets at the bottom of the test tube were washed 3 times with a 0.85% (*w*/*v*) sodium chloride (NaCl) solution to remove impurities and residual broth. The serum bottles (50 mL) were filled with 25 mL of sterilized MSM with a pH of 7. The inoculum size was considered as 2% (*v*/*v*), which was aseptically added to the media. A total of 2% (*v*/*v*) filter-sterilized dextrose was used as a carbon source. The serum bottles were sealed with a crimp cap and rubber stopper. Nitrogen gas was purged in bottles for 5 min to provide anaerobic conditions. All samples were incubated at 30 °C on a shaker at 150 rpm for 5 days. Agitation was applied to enhance contact between microbial cells and substrates while maintaining a homogeneous distribution of nutrients. The cell-free supernatants from this step were used in biosurfactant screening tests. The cell-free supernatants were achieved by centrifuging culture broth at 10,000 rpm for 15 min to obtain the cell pellets. The supernatants were collected and passed through a sterile 0.22 µm nylon syringe filter [20].

### 2.5. Biosurfactant Screening Tests

To determine biosurfactant producers among the bacterial isolates, various biosurfactant screening tests were performed. Amongst the various tests, oil displacement, blood agar, cetyl trimethyl ammonium bromide (CTAB) agar, and drop collapse assays were used [21]. The screening tests were performed in triplicate. According to these results, the most efficient strain was selected for deeper study with respect to the biosurfactant characterization.

#### 2.5.1. Oil Displacement

This is a rapid and quantitative method. A total of 40 mL of deionized water was poured into clean Petri dishes, and 20 μL of crude oil was added on the top to form a thin layer. Then, 10 μL of supernatant was placed on the oil surface. The formation of the clear zone has a direct relation to the biosurfactant concentration.

#### 2.5.2. Blood Agar Assay

Hemolysis activity was studied in 5% sheep blood agar. The lysis of blood cells creates a clear zone around the inoculation spots produced by the biosurfactant producers. Thus, 50 μL of culture broth was inoculated into holes made in the agar surface and incubated anaerobically at 25 °C for 48 h [22].

#### 2.5.3. Drop Collapse Test

This is a quick, qualitative method based on visual observation to assess the presence of biosurfactant in cell-free supernatants. For this assay, a single oil droplet was placed on a clean microscopic slide, and one drop of the cell-free supernatant was placed on top. After 1 min, if the supernatant formed a convex surface, it was considered a negative result; however, a flattened surface indicated a positive result. In this test, water was used as the negative result and Tween 80 as the positive result [23].

#### 2.5.4. Cetyl Trimethyl Ammonium Bromide (CTAB) Agar

This is a qualitative method for identifying anionic biosurfactant producers. Minimal salt media (Table 1) with 1.5% agar was prepared, and the pH was adjusted to 7 before autoclaving. The minimal salt media was supplemented with methylene blue (0.5 mg/mL) and CTAB (0.2 mg/mL). Holes were made on the agar surface by a cork borer and inoculated with each isolate. The plates were sealed with parafilm and placed in a jar for incubation at 30 °C for 24 h under anaerobic conditions (jar-and-pack method). In this test, the precipitation of the methylene blue in the presence of the CTAB and anionic surfactant creates a dark blue halo around each hole. An abiotic control was considered a negative result [24].

### 2.6. Identification of the Most Efficient Strain by 16s rRNA Gene Sequencing

Based on the screening results, the strain that showed the most effective results across all tests was selected for gene identification. A pure colony was grown overnight on an LB agar plate. A single colony was selected, heat-treated at 95 °C for 10 min in a thermocycler, and used as the template for PCR amplification. The PCR products were subsequently analyzed by agarose gel electrophoresis. A negative control was considered. The sequencing was performed by an external laboratory. Then, the obtained gene sequence was uploaded to the NCBI nucleotide blast search for strain identification. In addition, the phylogenetic tree was created using MEGA11 version 11.0.13, and FigTree version 1.4.4 was used to adjust the font and improve the tree’s visualization [23].

### 2.7. Biosurfactant Extraction

The culture broth was centrifuged at 10,000× *g* for 15 min at 4 °C. The cell-free supernatant was collected by passing it through a syringe filter (0.22 μm). The supernatant was gradually acidified to pH 2 by adding concentrated HCl (1N). The acidified solution was refrigerated at 4 °C overnight. Chloroform–methanol (2:1) was used for solvent extraction, and the extraction was repeated 3 times. Solvent evaporation was performed by rotary evaporation and a water bath at 40 °C. The crude biosurfactant was collected [25].

### 2.8. Determination of Critical Micelle Concentration (CMC)

The surface tension of the liquid decreases with increasing biosurfactant concentration until the CMC is achieved, where the biosurfactant forms micelles around a hydrophobic substance. Above the CMC, increasing the biosurfactant concentration results in no further decrease in surface tension. To evaluate the CMC point, a KRÜSS force tensiometer equipped with a Wilhelmy plate, at 25 °C, was used to measure the surface tension at biosurfactant concentrations ranging from 0 to 4 g/L. Each concentration was measured 5 times and the average obtained values (mN/m) were plotted in a graph using Excel software version 2603 [26].

### 2.9. Biosurfactant Characterization by Fourier Transform Infrared Spectroscopy (FT-IR) Analysis

In this experiment, dried biosurfactants were analyzed by the FTIR device (Bruker Optics, Germany, Invenio S). A spectrum was created based on the transmittance of the biosurfactant functional groups at specific wavelengths between 400 cm^−1^ and 4000 cm^−1^ [27].

### 2.10. Emulsification Index 24 (EI24)

The emulsification index of the most efficient strain was evaluated by substituting different carbon sources, including glycerol, whey, and dextrose. A control without a carbon source was considered. The MSM was supplemented with 4% carbon sources and 5% inoculum, and the mixture was incubated at 37 °C in a shaker incubator at 150 rpm for 5 days. Nitrogen gas was used to provide anaerobic conditions. To measure EI24, 2 mL of cell-free supernatant was vortex-mixed with various hydrocarbon sources, such as hexane, toluene, and kerosene, for 2 min. Then, it was left undisturbed at room temperature. After 24 h, the height of the emulsified layer was measured and divided by the total height [28].

### 2.11. DCPIP (2,6-Dichlorophenolindophenol) Test

The bacteria’s ability to degrade hydrocarbons was tested using the DCPIP method. This redox indicator loses its dark blue colour in the medium upon oxidation of the carbon source in the presence of the bacteria. Thus, 10 mL of sterilized MSM media with a pH of 7, supplemented with 1% (*v*/*v*) crude oil, 1% DCPIP, and 1% inoculum, was incubated on a shaker with a speed rotation of 150 rpm for 5 days anaerobically. The agitation was applied to ensure uniform dispersion of the substrate, particularly for hydrophobic compounds. The media colour was visually checked daily with an abiotic control [29].

### 2.12. Mature Fine Tailings Bioremediation

Bacterial isolates that passed positive biosurfactant screening tests (D4, D5, D6, and D11) were inoculated at a concentration of 5% (*v*/*v*) into 400 mL of sterilized MSM media and 400 mL of mature fine tailings. A control without inoculation, including the media and the mature fine tailings, was also considered. For this experiment, 1000 mL Pyrex flasks (Fisherbrand™, Ottawa, ON, Canada) with open-top screw caps and silicone septa were used. The bottles were sealed and purged with nitrogen gas for 20 min to create anaerobic conditions. The samples were stored at room temperature away from sunlight; samples were collected weekly, with the following factors measured: viability of the bacteria, surface tension, pH, anion concentration, hydrocarbon degradation, CO_2_, and CH_4_ production.

#### 2.12.1. Surface Tension Measurement

The surface tension of the MFTs was directly measured by Fisher Scientific Tensiometer model 21 (Fisher Scientific Ottawa, ON, Canada), employing a Du Nouy ring method at room temperature in triplicate.

#### 2.12.2. pH Measurement

The pH of the sample was measured by a pH benchtop (Thermo Scientific, Model Orion 2 start, Ottawa, ON, Canada) at room temperature in triplicate.

#### 2.12.3. Viability of the Bacteria

Bacterial viability was measured as colony-forming units (CFUs) per milliliter (mL) of the sample. One milliliter of the MFTs was serially diluted to 10^−8^ and plated on LB agar. The Petri dishes were sealed with parafilm, and the samples were incubated anaerobically using the jar-and-pack technique at 30 °C for 24 h. Numbers of cells were counted visually in triplicate [30].

#### 2.12.4. Hydrocarbon Degradation Analysis

The concentration of the hydrocarbons was measured using an Agilent 8990 gas chromatography–flame ionization detection (GC-FID) instrument (Agilent Technologies Co., Ltd. Santa Clara, CA, USA). The hydrocarbons were extracted via mechanical shaking with dichloromethane (DCM) according to a previously reported method [31]. The GC-FID method was developed according to the study of Yang et al. [32]. The standard contained n-alkanes ranging from C9 to C30 (Sigma-Aldrich Canada Ltd., Oakville, ON, Canada). Hydrocarbon degradation was calculated as a percentage relative to the initial MFT concentration, and was calculated according to the following equation:

Hydrocarbon degradation (%) = C_0_ − C_t_/C_0_ × 100% (1)

where C_0_ = initial hydrocarbon concentration; and C_t_ = hydrocarbon concentration at time t.

#### 2.12.5. Gas Production (CO_2_, CH_4_)

Samples were collected from the headspace of the bottles weekly using a gas-tight syringe (Hamilton Company, Reno, NV, USA) and manually injected into a gas chromatograph (Agilent 7890A GC system, Santa Clara, CA, USA) equipped with flame ionization (FID), thermal conductivity (TCD), and electron capture (ECD) detectors, operated using ChemStation software version B.04.03). The temperature program was set to 40 °C as the initial oven temperature, held for 2 min, then increased from 40 to 150 °C at 20 °C/min and held at 150 °C for 2 min to clean the column. The total operation time lasted for approximately 8 min.

### 2.13. Statistical Analysis

All experiments were performed in triplicate, and the results are presented as mean ± standard deviation where applicable.

## 3. Results

### 3.1. Isolated Bacterial Strains from MFT

A total of 12 bacterial colonies were isolated on different agar plates. The results showed that seven of the colonies were isolated on minimal salt agar, four colonies formed on R2A agar, and one colony was successfully isolated on Bushnell–Haas agar under anaerobic conditions. All the strains exhibited a clear zone after incubation, demonstrating their tolerance to growth in crude oil or oil consumption. Table 2 shows the colony characterizations.

### 3.2. Results of Biosurfactant Screening Tests

Each isolate was subjected to various biosurfactant screening methods (e.g., blood agar, CTAB medium agar, oil displacement and drop collapse). Table 3 shows the summarized results. As shown in the table, isolated D11, D6, D5, and D4 had better results than the others. The blood agar medium test was positive for D11, D4, D5, D6 and MSA8. Several studies have used this method to isolate biosurfactant producers [33,34]. Moreover, this strain showed positive results on CTAB agar, confirming its production of an anionic biosurfactant. Many studies used this method as a selective agar for identification of anionic biosurfactant; however, the CTAB compound in this media might have inhibitory effects on some microorganisms [35]. Furthermore, strain D11 showed a positive drop collapse, and, through oil dispersion assays, strain D11 showed the greatest oil dispersion, with a 2.4 cm diameter, compared to other strains; as a result, it was selected as the most efficient strain. Biosurfactant production and characterization of this strain were selected for further study.

### 3.3. 16s rRNA Gene Sequencing and Phylogenetic Results

**Figure 1:**
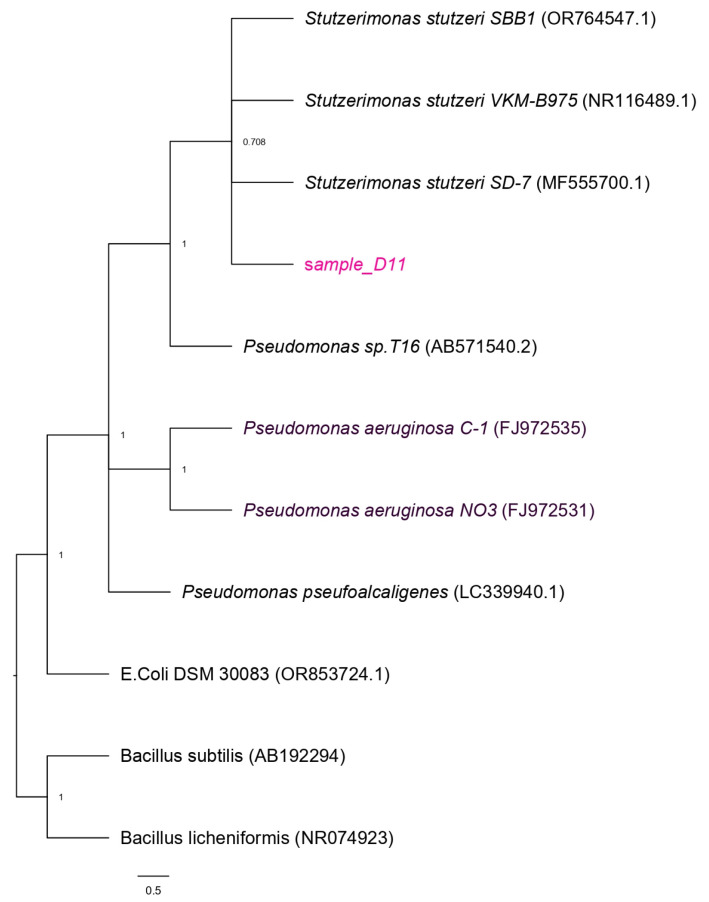
Phylogenetic tree of sample “D11”.

The obtained gene sequence was deposited in the NCBI GenBank database, and online BLAST analysis in NCBI website revealed that isolate D11 showed 100% sequence similarity to *Stutzerimonas stutzeri* strain SD-7 (accession number MF555700.1), indicating that the isolate is closely related to *Stutzerimonas stutzeri*. This strain is facultative anaerobic bacteria belonging to class Gammaproteobacteria, meaning that this strain is metabolically flexible and, based on the environmental conditions, uses oxygen or other electron acceptors for growth. This strain is a motile Gram-negative bacterium with positive oxidase and catalase tests. Figure 1 shows a phylogenetic tree that clusters isolate D11 with its closest relatives, with *Pseudomonas* species identified as the nearest neighbours. The tree was rooted with *E. coli* and *Bacillus* species. The node values indicate high confidence in species grouping. A phylogenetic tree was constructed using the neighbour-joining method and the Kimura two-parameter model. Branch support was assessed using a bootstrap analysis with 1000 replicates.

### 3.4. Surface Tension and CMC Evaluation

**Figure 2:**
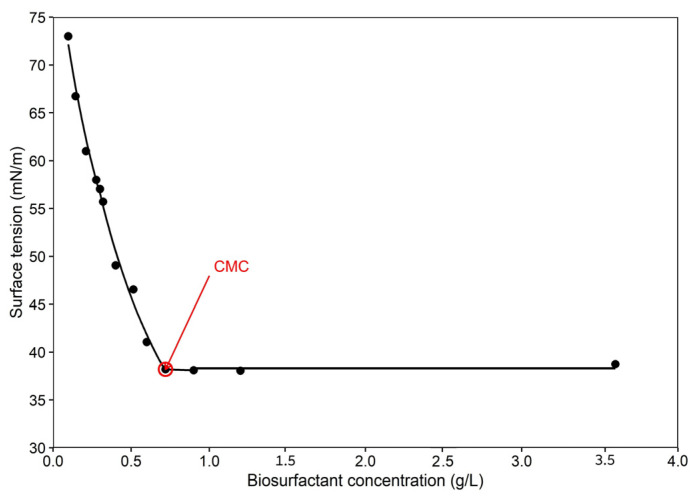
CMC curve of produced biosurfactant by *Stutzerimonas stutzeri*.

The CMC of the produced biosurfactant by strain *Stutzerimonas stutzeri* was determined based on the correlation between the biosurfactant concentration and surface tension value. The CMC value of the crude biosurfactant is demonstrated in Figure 2. The CMC point for this strain was indicated to be 600 mg/L, where the surface tension value decreased from 72 ± 0.02 mN/m to 38.70 ± 0.08 mN/m. The CMC value depends on various factors such as environmental conditions, strain, biosurfactant purity and the chemical structure of the biosurfactant [36]. The biosurfactant yield when cultivating this strain with dextrose as a carbon source showed 2.1 g/L under anaerobic conditions.

### 3.5. Emulsification Index 24 (EI24)

The *Stutzerimonas stutzeri* emulsification index (EI24) was affected by different carbon sources, resulting in varied emulsification values for various hydrocarbons. The culture broth supplemented with whey as a carbon source exhibited the highest emulsification index (EI24) against toluene (24%), hexane (60%), and kerosene (46%), respectively. The EI24 of bacteria cultivated in media supplemented with glycerol exhibited the greatest emulsification with kerosene (53%), hexane (53%), and toluene (26%). The lowest EI was observed with dextrose as the carbon source in the media for kerosene (33%), toluene (26%), and hexane (46%). The difference in EI24 is due to the formation and composition of the biosurfactant produced when consuming different carbon sources. It can also be concluded that using agro-industrial waste (whey and glycerol) as a substrate promotes the formation of stable emulsions toward different hydrocarbons, especially complex and aromatic ones.

### 3.6. Biosurfactant Characterization by FTIR

**Figure 3:**
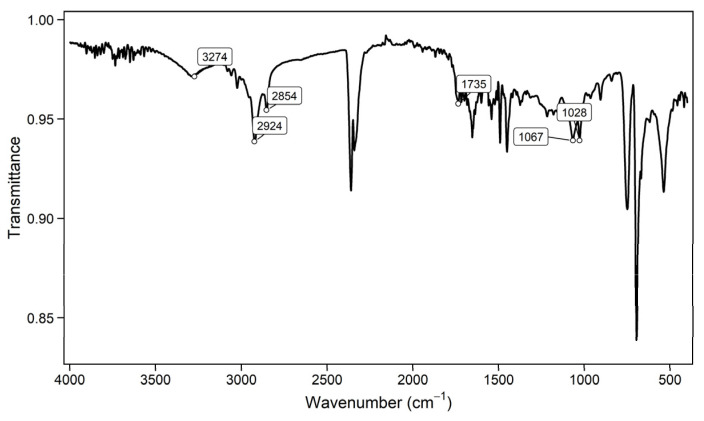
FT-IR spectrum.

FTIR spectroscopy was used to reveal the major functional groups of the extracted biosurfactant. The FTIR spectra, based on peaks commonly reported, suggest a glycolipid type of rhamnolipid biosurfactant (Figure 3). Rhamnolipid consists of a rhamnose ring that connects an ester group to a fatty acid chain. The peaks at 1028 and 1067 cm^−1^ are related to the C-O functional group. Another essential peak occurs at 1735 cm^−1^. This peak is common among rhamnolipid-type biosurfactants. It indicates the C=O stretch. This stretch links the ester group to the fatty acid chain. The lengths of the fatty acid chain with symmetric and asymmetric bonds between CH_2_-CH_3_ are demonstrated by peaks at 2854 and 2924 cm^−1^. The O-H stretch is defined by the hydroxyl group, and its related transmittance is around 3274 cm^−1^. These essential peaks suggest a glycolipid–rhamnolipid classification with respect to biosurfactants. Previous studies have also reported rhamnolipid production by the strain by *Stutzerimonas stutzeri* [37,38].

### 3.7. DCPIP Test

A DCPIP test was performed on all isolates. Among isolates, *Stutzerimonas stutzeri* (strain D11) showed a positive result by reducing the dark colour of the redox indicator under anaerobic conditions, while the abiotic control remained unchanged after incubation. These findings suggest that this strain can actively degrade hydrocarbons under anaerobic conditions. This finding aligns with prior research in which *S. stutzeri* was cultivated on pyrene with DCPIP serving as the electron acceptor [39].

### 3.8. Biodegradation of Mature Fine Tailings

#### 3.8.1. Assessment of Live Bacteria

**Figure 4:**
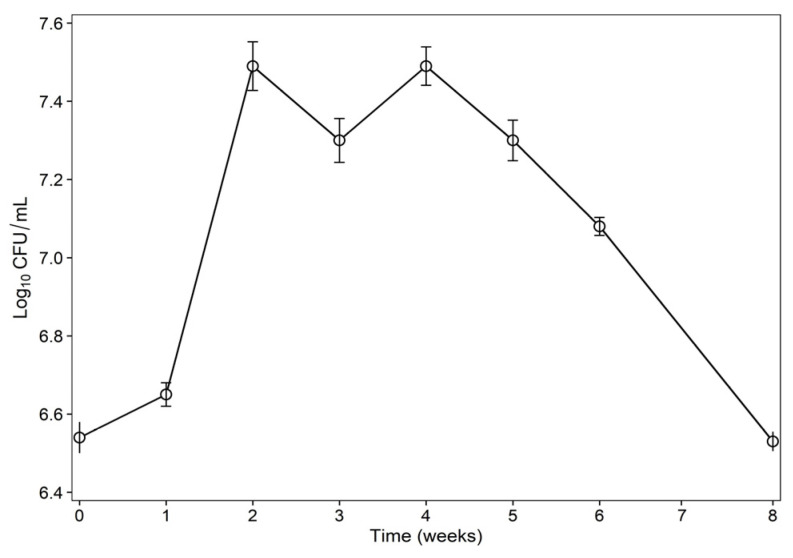
Bacteria population over time.

The growth of the bacterial population (sample 1) during 8 weeks of anaerobic incubation is shown in Figure 4. Sample 1 included equal amounts of unsterilized MFTs and MSM that were inoculated with a consortium of isolates (D4, D5, D6, and D11) that were positive in biosurfactant production assays. The bacterial count remained almost the same in the first week of the experiment. This stage represents the lag phase, during which the consortium adapts to the environmental conditions of the batch experiment. There is a substantial increase in the number of live bacteria in week 2. The stationary phase appears from week 2 to week 4, during which the number of bacteria remains almost the same. A small fluctuation in week 3 might be due to the presence of complex microorganisms with different lag phases in MFTs. A final phase occurs from week 5 to week 8. As nutrient depletion occurs in the system, the population of bacteria decreases.

#### 3.8.2. Changes in Surface Tension During Bioremediation of MFT

**Figure 5:**
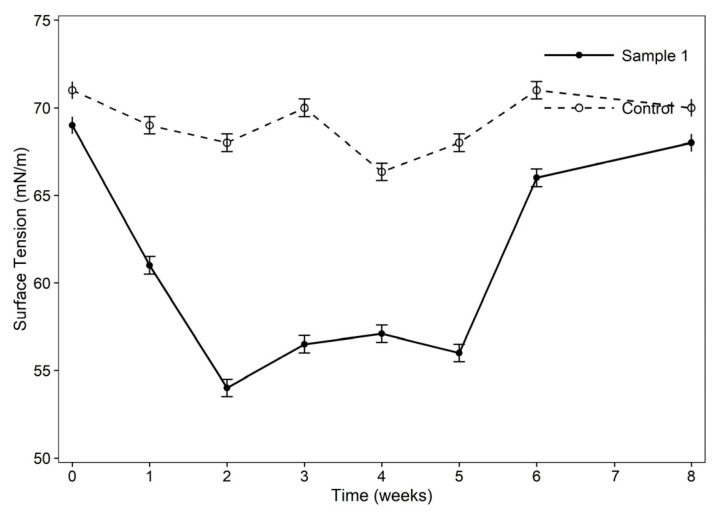
Surface tension comparison of the sample with the control.

Surface tension was monitored to assess biosurfactant production from the inoculated bacterial consortium and compared with the uninoculated control. The changes in surface tension were compared between sample 1 and the control over time (Figure 5). The initial surface tension of the MFTs was 69 ± 0.5 mN/m, decreasing to 54.33 ± 0.5 mN/m in week 2 under anaerobic conditions, which aligns with the exponential growth of the bacterial population and the increase in biosurfactant production. This value showed minor fluctuations until week 5, which is consistent with the stationary phase of bacterial growth. However, this value again increased to (67.66 ± 0.5 mN/m). The reason might be related to the natural decomposition of the biosurfactant produced due to nutrient deficiency by bacteria. The control sample consisted of unsterilized MFTs, which showed a surface tension factor that was almost the same throughout the experiment, ranging from 70 ± 0.5 mN/m to 66 ± 0.5 mN/m.

#### 3.8.3. pH Variation During MFT Bioremediation

**Figure 6:**
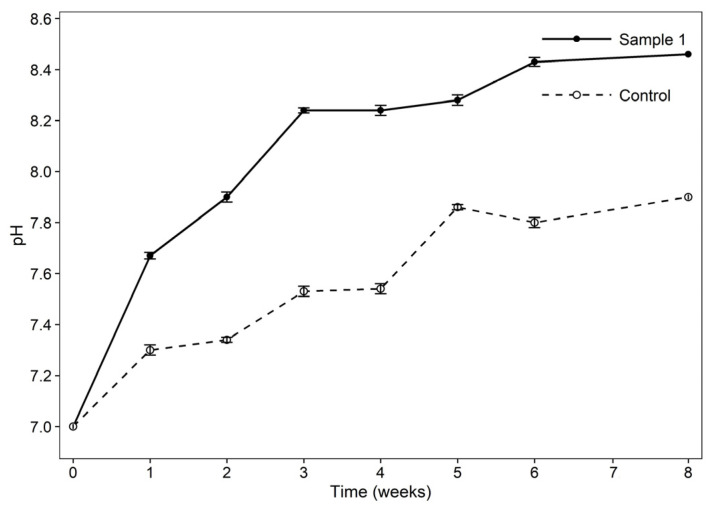
pH changes over time for sample 1 and the control.

The pH of the MFTs significantly increased from 7 ± 0.2 at the beginning of the experiment to 8.24 ± 0.1 in week 3 and then rose gradually to 8.46 ± 0.1 at the end of the experiment (Figure 6). The increase in pH is due to bacterial metabolic activity and its chemical by-products, especially during weeks 2 and 3. *Stutzerimonas stutzeri* is a denitrifying strain that uses a readily available nitrate source (NaNO_3_) as an electron acceptor and hydrocarbons as electron donors for oil degradation. This process results in the production of OH^−^, which causes an increase in media pH [40]. The control sample showed an initial pH of 7.0 ± 0.1, and at the end of the experiment, the pH changed to 7.86 ± 0.1. The same pattern was observed in previous studies investigating the effect of denitrifying bacteria on pH increases under anaerobic conditions within the first few days of their activity. Alkalinity is generated from hydroxide ions during this process [41]. Consequently, active denitrification increases pH during the bioremediation of hydrocarbons in the presence of the biosurfactant.

#### 3.8.4. Hydrocarbon Degradation During Bioremediation of MFT

**Figure 7:**
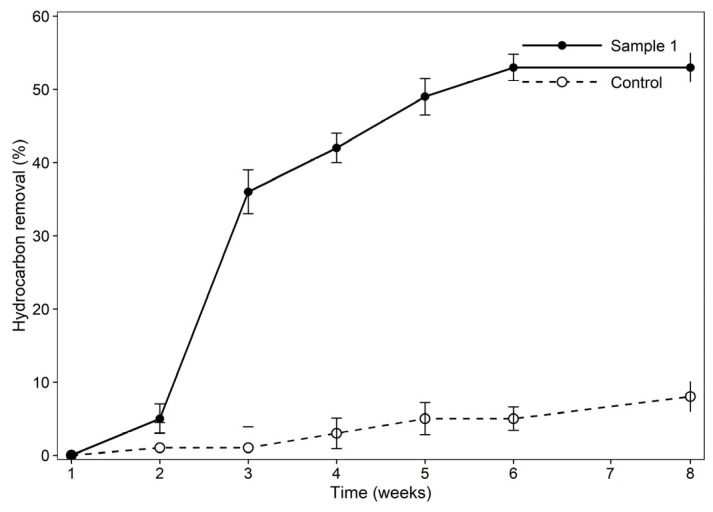
Hydrocarbon removal of sample 1 and the control.

The hydrocarbon removal differed between the treated batch (sample 1) and the control (Figure 7). Sample 1 showed 53% hydrocarbon removal under anaerobic conditions over 6 weeks of the experiment. The lag phase of removal during the first 2 weeks of incubation is common and may be related to microbial acclimation and limited bioavailability of hydrocarbons to bacteria, especially under anaerobic and static conditions [42]. However, from week 2 to week 3, the concentration of n-alkane hydrocarbons decreased by 36%, while the control remained below 3%. This sharp increase suggests active biodegradation of the hydrocarbons by bacteria and marks the late exponential and the onset of the stationary growth phase. During that time, the production of the biosurfactant and a reduction in surface tension were also observed. Biodegradation gradually increased to 53% by week 6, and thereafter no further decrease in hydrocarbon removal was observed, which may be due to nutrient limitation and reduced bioavailability of the hydrocarbons. The control sample showed a modest 8% increase in degradation, which is attributed to slow indigenous bacterial activity rather than active degradation over the time. According to the results, the presence of biosurfactant producers accelerated hydrocarbon degradation by more than 6 times compared to the control. Similarly, a previous study by Chaudhary et al. [43] applied the biostimulation method for hydrocarbon degradation, which led to (<25%) limited hydrocarbon degradation, while combining the biostimulation (nutrient amendment) and bioaugmentation could result in 98% of hydrocarbon removal in 60 days. The key to their success in research was an optimized C:N:P ratio of 100:10:1, along with the addition of five isolated indigenous bacterial populations under optimized conditions, which led to highly efficient removal. These findings suggest that biostimulation alone is insufficient for hydrocarbon removal in systems lacking sufficient active indigenous bacteria [43]. Previous studies on the biodegradation of crude oil by strain *Stutzerimonas stutzeri* under optimal conditions achieved 77.9% under aerobic conditions [44]. In another study, the crude oil removal efficiency of this strain under aerobic conditions was reported as 84% under optimized conditions, and enzymes such as alkane hydroxylase, alcohol dehydrogenase, and laccase were identified as degradative enzymes [45].

#### 3.8.5. Assessment of CO_2_ and CH_4_ Production

**Figure 8:**
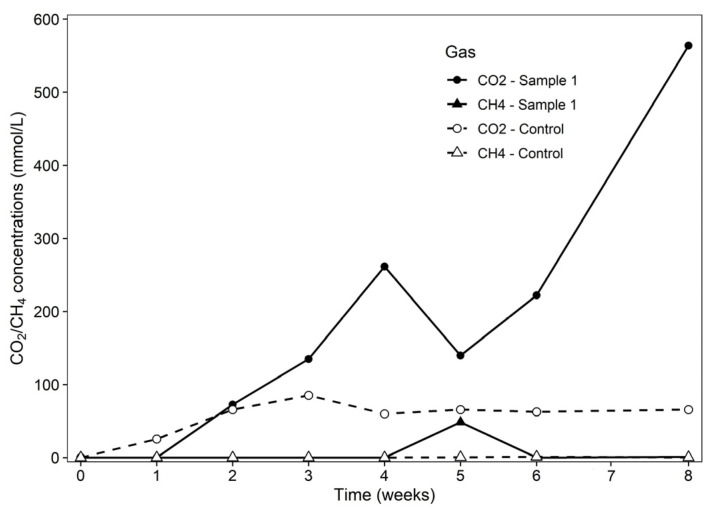
Sample 1 and control production of CO_2_ and CH_4_.

The generation of CO_2_ and CH_4_ during incubation was measured to evaluate the bioremediation of hydrocarbons under anaerobic conditions. Figure 8 compares the methane and carbon dioxide production for sample 1 and the control. In the first week, CO_2_ production is negligible for both sample 1 and the control. The gradual increase begins in week 1 and continues through week 4, from 72.44 (mmol/L) to 261.55 (mmol/L), corresponding to the end of the bacteria’s stationary phase. During this time, methane production is inhibited by the dominant denitrifying bacterial population, and hydrocarbon degradation peaks and surface tension decrease. There is a syntrophic association between methane and carbon dioxide in week 5, suggesting the activation of methanogenic bacteria. In the last two weeks, the exponential increase in carbon dioxide production without further hydrocarbon removal suggests the degradation of organic substances, such as dead cells or humid compounds within the MFTs, or the incomplete degradation of hydrocarbons rather than complete mineralization [46,47].

## 4. Discussion

This laboratory-scale study focused on isolating native bacteria from mature fine tailings under anaerobic conditions, as well as performing biosurfactant screening tests to evaluate potential bacteria for biosurfactant production. Additionally, among the isolates, only the most effective strain was selected for molecular identification and biosurfactant characterization, including determination of biosurfactant yield, critical micelle concentration (CMC), identification of the functional group of the produced biosurfactant by FT-IR, and EI24 toward various hydrocarbons. The results of the biosurfactant production were consistent with previous studies reporting that indigenous bacteria isolated from hydrocarbon-contaminated sites can produce biosurfactants, which enhance hydrocarbon bioavailability [13,34,47,48]. Bioremediation of the tailings was examined by reintroducing the consortium of biosurfactant producers of native bacteria from mature fine tailings into the waste with minimal salt media supplementation. Throughout this process, key parameters such as the survival of the bacteria, chemical changes such as pH, biogas production, and hydrocarbon removal were monitored. The finding of bioremediation of mature fine tailings by microbial consortia aligns with the successful application of bacterial consortium to contaminated environments, which has been previously reported to be more effective than monocultures due to various factors such as the diversity of metabolic interactions and substrate consumption [49,50,51].

Moreover, these experimental findings revealed that bioaugmentation with indigenous bacteria from mature fine tailings is a promising technique for anaerobic hydrocarbon degradation. This method can be practical in sites with low bacterial populations or inactive indigenous bacteria. The use of native bacteria leads to faster bioremediation, as these bacteria are adapted to the harsh environment of the waste such as limited nutrients and oxygen and, in general, they demonstrate that they can survive in the presence of co-contaminants. Similar studies have demonstrated higher degradation efficiency in oil-polluted environments by native bacteria rather than exogenous cultures [52].

In addition, the experimental set up is designed to represent conditions present in the mature fine tailings layer in a tailings pond by maintaining the anaerobic conditions and avoiding mechanical agitation.

Despite these promising results, this study has several limitations that should be acknowledged. First, the study focused on a limited number of bacterial isolates, which may not have captured the full microbial diversity of MFTs. Future studies should investigate the microbial community with a focus on gene analysis for a better understanding of the role of different microorganisms in hydrocarbon degradation. Moreover, long-term stability in natural field conditions and seasonal temperature changes were not considered. For practical field applications, it may be beneficial to investigate bacteria that remain metabolically active in lower temperatures, particularly those adapted to cold regions. In addition, creating a kinetic model would greatly help to reach a better understanding of the biodegradation dynamics present in the system. For example, modified Monod-type models that incorporate hydrocarbon bioavailability factors could improve insight into microbial degradation processes. More specifically, coupling biodegradation kinetics with mass transfer models allow for a more accurate representation of the multiphase nature of the tailings matrix.

## 5. Conclusions

Biosurfactants have appeared as a green, sustainable technique for the remediation of various environmental pollutants, especially in the oil and gas sector, where persistent, toxic waste requires specialized measures. This study examined native bacteria from mature fine tailings to assess their potential for biosurfactant production. The finding showed that among 12 bacterial isolates, four produced biosurfactants. The most effective strain was selected for gene sequencing. Results showed the highest sequence similarities to *Stutzerimonas stutzeri*, and its biosurfactant was characterized. The results showed that this strain can produce rhamnolipids under anaerobic conditions. A biosurfactant yield of 2.1 g/L was achieved at 37 °C on an orbital shaker at 150 rpm, using 4% dextrose as the carbon source and 5% inoculum. The CMC point was determined to be 600 mg/L. Moreover, this strain has demonstrated strong emulsification of both simple and complex hydrocarbons when cultivated in MSM supplemented with low-cost carbon sources such as whey and glycerol. This suggests the potential of this strain for consuming agro-industrial waste sources for biosurfactant production. MFTs bioremediation was conducted by the bioaugmentation method. The bacterial consortium could reduce n-alkane hydrocarbons in waste by 53% over 6 weeks of incubation at room temperature. Meanwhile, the surface tension of the media decreased from 69 ± 0.5 mN/m to 54.33 ± 0.5 mN/m due to active bacterial surfactant production. Thus, the results of this study suggest that this method could effectively reduce n-alkane levels and that bioaugmentation with native bacteria could shorten the lag phase of bacterial growth, thereby accelerating biodegradation. In conclusion, this study’s results indicate a positive outcome for biosurfactant production from indigenous bacteria in mature fine tailings. Additionally, the results of the hydrocarbon degradation suggest its application in the in situ bioremediation of hydrocarbon contamination.

## Figures and Tables

**Table 1:** Minimal salt media (MSM) composition.

Chemical Name	Concentration (mg/L)
NaNO_3_	2000
MgSO_4_	100
CaCl_2_·2H_2_O	33
KCl	500
KH_2_PO_4_	1000
Na_2_HPO_4_H_2_O	1000
FeSO_4_·7H_2_O	1
Trace metals:	2 mL/L
FeCl_3_·6H_2_O	60
Na_2_MoO·4H_2_O	15
H_3_BO_3_	150
CoCl_2_·6H_2_O	60
CuSO_4_·5H_2_O	590
ZnSO_4_·7H_2_O	600
MnSO_4_·H_2_O	200

**Table 2:** Morphological colony characterization.

Strain Name	Colony Colours	Colony Shape
MSA1	Transparent	Small
MSA2	White	Creamy irregular
MSA5	Green	Moderate circle
MSA7	White	Small
MSA8	Green	Small circle
MSA9	White	Moderate circle
MSA10	Brown	Small circle
BH1	Green	Small circle
D4	White	Moderate circles
D5	Yellow	Moderate circle
D6	Yellow	Small circle
D11	White to brownish	Dry wrinkle edge

**Table 3:** Biosurfactant screening results.

Strain Name	Blood Agar Medium	CTAB Medium	Oil Displacement	Drop Collapse
MSA1	−	−	−	−
MSA2	−	−	−	−
MSA5	−	−	−	−
MSA7	−	−	−	−
MSA8	+	−	+	+
MSA9	−	+	−	−
MSA10	−	+	−	−
BH1	+	−	+	+
D4	+	+	+	+
D5	+	+	+	+
D6	+	+	+	+
D11	+	+	+	+

“+” means a positive result; “−” means a negative result.

## Data Availability

Data is contained within the article.
